# Challenges for Economic Evaluation of Health Care Strategies to Contain Antimicrobial Resistance

**DOI:** 10.3390/antibiotics8040166

**Published:** 2019-09-27

**Authors:** Emily A. F. Holmes, Dyfrig A. Hughes

**Affiliations:** Centre for Health Economics and Medicines Evaluation (CHEME), Bangor University, Normal Site, Bangor, Gwynedd LL57 2PZ, UK; d.a.hughes@bangor.ac.uk

**Keywords:** antimicrobial resistance, economic evaluation, cost-utility analysis, cost-effectiveness analysis, antibiotics

## Abstract

The threat of antimicrobial resistance has global health and economic consequences. Medical strategies to reduce unnecessary antibiotic prescribing, to conserve the effectiveness of current antimicrobials in the long term, inevitably result in short-term costs to health care providers. Economic evaluations of health care interventions therefore need to consider the short-term costs of interventions, to gain future benefits. This represents a challenge for health economists, not only in terms of the most appropriate methods for evaluation, but also in attributing the potential budget impact over time and considering health impacts on future populations. This commentary discusses the challenge of accurately capturing the cost-effectiveness of health care interventions aimed at tackling antimicrobial resistance. We reflect on methods to capture and incorporate the costs and health outcomes associated with antimicrobial resistance, the appropriateness of the quality-adjusted-life year (QALY), individual time preferences, and perspectives in economic evaluation.

## 1. Introduction

Antimicrobial resistance encapsulates the loss of effectiveness of any anti-infective medicine, including antiviral, antifungal, antibacterial, and antiparasitic medicines [1,2]. The threat of antimicrobial resistance has global health and economic consequences; resistant infections are responsible for an estimated 700,000 deaths annually worldwide which, if no action is taken, could result in a cumulative cost of $100 trillion by 2050 [3]. Urgent threats include *Clostridioides difficile*, carbapenem-resistant *Enterobacteriaceae* (CRE), and drug-resistant *Neisseria gonorrhoeae* [4]. The current rate of *Clostridioides difficile* infection in the United States (US) alone is 500,000 cases and 15,000 deaths per year. The urgency of addressing antimicrobial resistance is recognised widely [4,5], and a range of containing strategies have been implemented, including antimicrobial stewardship, which has become a central aspect of delivering safe and effective health care [6]. Antimicrobial stewardship involves a coordinated approach to promote and monitor the judicious use of antimicrobials to preserve their future effectiveness [6]. The UK’s five-year action plan outlines three key targets for tackling antimicrobial resistance: (i) reducing need and unintentional exposure, (ii) optimizing use, and (iii) investing in innovation, supply, and access [3]. A key strategy to achieve these aims includes more appropriate prescribing, which includes reducing unnecessary antibiotic prescribing [7,8,9,10]. Not only is unnecessary antibiotic prescribing costly for little or no therapeutic benefit, it places patients at risk of adverse effects [8,11] and contributes to the development of antibiotic resistance [7,8,9,10]. The potential for improved health outcomes and lower costs are easily recognised; however, health care strategies may also include trade-offs between short-term outcomes and long-term gains, for example, not using the most effective anti-infective medicine, in order to preserve future effectiveness. This represents a shift of focus of evaluation, from clinical decision making for an individual patient in the present, to a longer-term (intergenerational) public health agenda. Similarly, there has been a shift of focus in the pharmaceutical industry, with strategies incentivizing the pharmaceutical industry to develop new drugs, using funding mechanisms not linked to volume [3].

Economic evidence is central to understanding the value of competing health care strategies to lessen the probability of resistance development [12,13]. Interventions or services are typically evaluated using standard methods of health technology assessment, including cost-effectiveness or cost-utility analyses. Economic evaluations inform judgements on whether the value of additional health benefits exceed the opportunity cost; where opportunity cost is the next best alterative foregone. As conventionally applied, health benefits relate to the effectiveness of interventions in relation to managing infections less any adverse effects, which are typically confined to adverse drug reactions. However, the application of such methods in the context of antimicrobial resistance poses several unique methodological challenges, not least in quantifying the costs and externality effects (impacts on other patients) of future reductions in antimicrobial effectiveness (Table 1).

## 2. Challenges

### 2.1. Capturing the Benefits of Strategies to Contain Antimicrobial Resistance

How best to assess the value of any intervention to reduce antimicrobial resistance is a methodological challenge, which requires adequate measurement of the expected rate of growth of antimicrobial resistance and associated outcomes over time [15]. Economic evaluation of health care strategies to reduce antibiotic prescribing, for instance, ought to value their impact on antimicrobial resistance. Most economic evaluations in this area, however, fail to consider the costs and outcomes relating to antimicrobial resistance—or where they have, consideration has been restricted to projected financial costs and was not reflective of population health [16,17]. In order to achieve this, data are required on the long-term health outcomes of both current patients and future cohorts of patients who may be prescribed antimicrobial therapy in the future. This includes the health outcome of patients who become infected by a resistant pathogen, or who have not experienced resistant infection but receive alternative agents due to increased resistance of common pathogens. The disutility of potentially less effective treatment represents an important economic parameter that is required in addition to the capturing the more obvious morbidity and mortality associated with short-term health outcomes of the patient presenting with an infection.

#### Measure of Health Outcome

Economic evaluations of health care interventions to tackle antimicrobial resistance may consider different measures of health outcomes, ranging from the number of prescriptions avoided, to years of life gained and quality-adjusted-life years (QALYs). Cost-utility analyses, which yield incremental costs per QALY gained, measure value in terms of health utility and survival. Utility represents the value or desirability of a given health state, most typically based on indirect, multi-attribute, preference-based measures, such as the EQ-5D [18,19]. The appropriateness of QALYs for use in evaluation of acute conditions, such as respiratory tract infections, a common focus of intervention aimed at tacking antimicrobial resistance, has both measurement and evaluation problems. Health state utilities used to calculate the QALY are typically elicited using trade-off exercises, and the issue with acute infection is that when trading quality and quantity of life, the ill-health state may be perceived as transient, thus questioning the reliability of this method [20]. Furthermore, health care strategies to contain antimicrobial resistance—by design—are only unlikely to concern the outcomes of today. The valuation of future health outcomes is also important and represents a challenge for health economists. An alternative evaluative framework may be required to capture the complexity of health outcomes over time.

The disability-adjusted-life year (DALY) represents an alternative health index used in cost-effectiveness analyses. The DALY captures the number of healthy years lost, by incorporating reduction in life expectancy with years lost to disability; as such health care interventions seek to avert the DALY (as opposed to increasing the QALY). The DALY provides an estimate of the burden of disease, such as infectious diseases, that is useful in global health prioritization. Cassini and colleagues reported the DALYs caused by five infections with antibiotic-resistant bacteria across populations in Europe and found this to be substantive compare to other infectious diseases [21]. From a global health perspective, the DALY is an oft-used measure that can be utilised in economic evaluations.

Cost-benefit analysis (CBA) is an approach to economic evaluation that places a monetary valuation on the consequences as well as the costs of interventions. This may be achieved using methods such as willingness to pay, which has been suggested as an alternative to the QALY when evaluating acute disease [20]. The willingness to pay method is based on contingent valuation to elicit monetary value for items not typically traded, such as health, and therefore accounts for both health and non-health effects. Whilst this is considered more comprehensive, in many jurisdictions patients may be unfamiliar with purchasing health care directly, and this may have an impact on the reliability of the methods. A further challenge is the potential association with ability to pay, based on income and wealth. In the context of health care strategies to tackle antimicrobial resistance, however, cost-benefit analysis offers a feasible method for capturing the cost of the negative externalities, on a global scale, to represent the true value future benefits. Where applied with robust formative work, there is potential for this method to be used to elicit individuals’ willingness to pay to avoid an illness (resistant infection) or obtain the benefits of a future treatment.

### 2.2. Perspective

Prior to conducting any economic valuation, the perspective needs to be defined [22]. The perspective of the valuation, e.g., whether it is conducted from the point of view of the patient, health care payer, or society, will determine the costs and outcomes that need to be included in the analysis. In the UK, the National Institute for Health and Care Excellence (NICE) recommends the perspective on outcomes should be all direct health effects, whether for patients or other people; and the perspective adopted on costs within its Technology Appraisal programme should be that of the National Health Service (NHS) and personal and social services (PSS) [23]. Benefits are the assigned general population’s valuation of health outcome (obtained from surveys), and costs include the cost of treatment and associated health service resource use (e.g., GP visits, hospitalisation, etc.). Whilst this provides a reference case for comparison of different health technologies, such Technology Appraisals consider individual patients or cohorts of patients, whereas antimicrobial resistance should consider populations. Evaluation of containment strategies may necessitate a broader perspective, and evidence in other areas suggests that assessment of the same strategy from different perspectives can arrive at different conclusions [24].

The broadest perspective adopted in economic evaluation is the societal perspective. This should reflect the full range of social opportunity costs associated with different interventions [22], for example, productivity losses due to patients’ inability to work. However, there is a paucity of evidence on the long-term indirect impact of antimicrobial resistance on costs and outcomes [25], and this represents a significant challenge for health economists evaluating containment strategies. Economic evaluations with restrictive perspectives have potential to overestimate the value of interventions or services. Where the perspective for costs is limited to hospital or health service, indirect costs to current and future patients, such as working days lost due to resistant infection, or treatment toxicity, fail to be captured.

To fully assess the cost-effectiveness of strategies to contain antimicrobial resistance and to avoid the inefficient allocation of scarce health service resources, decision makers require economic evaluations that incorporate costs and outcomes beyond the patient and hospital and consider the full ramification of antimicrobial resistance. Defining the “society” then becomes a challenge within itself, as antimicrobial resistance impacts populations that extends across jurisdictions. Adoption of a global multiagency perspective represents new territory and a limitation of conventional health technology assessment (HTA) and health-economic approaches. In the absence of long-term data, economists have taken a pragmatic approach; for example, our evaluation of C-reactive protein testing to guide antibiotic prescribing for lower respiratory tract infection in Wales, included a sensitivity analysis of the cost of the long term global cost of antibiotic resistance [17]. A challenge for future economic evaluation is to incorporate more robust measurement and modelling of longer-term indirect costs and outcomes—that go beyond today’s cohorts of patients to consider the population of tomorrow.

### 2.3. Capturing the Long-Term Costs of Antimicrobial Resistance

The resource implications of antimicrobial resistance are most likely to accumulate in the long-term. Short-term, direct health effects may only include the effect of the intervention on the affected patient; however, longer-term effects on both the patient and others are more likely. It is therefore important that all costs are captured and incorporated into economic evaluations across adequate time-horizons. Whilst interventions to reduce unnecessary prescribing in a clinical setting may represent a short time horizon, e.g., prescribing outcomes and re-consultation over 28-days, the longer-term effects of unnecessary antibiotic prescribing extend to current and future patients’ lifetimes [10,26]. Initially, resource use and costs associated with interventions that aim to contain resistance would typically be captured using health records and/or patient reports in the short-term. Estimating long-term direct and indirect cost and the cost consequences to others, however, may present more of a challenge. The cost impact has potential to go beyond the lifetime horizon of the patient and impact the population of tomorrow, and in some situations the product life-cycle [27] may be more relevant than the life time of the patients being treated.

Where long-term costs have been considered in existing economic evaluations, these have relied on estimates of per-prescription costs of antimicrobial resistance based on rudimentary calculations of the global costs of resistance divided by the annual number of prescriptions in each geographic region [16]. Oppong et al. 2013 [16], reported three estimates for the cost of antibiotic resistance, based on the annual cost of resistance in the US ($55 billion) [28], the cost of multidrug resistance in the European Union (EU) (€1.5 billion) [29], and the cost of global resistance over a 35-year period (US$2.8 trillion annually) [5]. This method assumes that antibiotic prescribing is the main cause of resistance, but whilst more robust estimates are required, it is widely acknowledged that this would require complex modelling methods [15]. Robust estimates of other long-term health conditions, such as increased risk of *Clostridioides difficile* infection, also require consideration. Indirect costs are also often difficult to estimate, as they may bias against people who are not in employment and may fluctuate over time.

A further challenge is that both direct and indirect costs may be attributable to different systems and payers at different time points. For example, even when restricting analysis to direct health service costs, the initial outlay for point of care C-reactive protein testing to avoid unnecessary antibiotic prescribing may be borne by a local health service provider; however, the longer-term cost savings are likely to be at a secondary level. Data collection should not be restricted to a single source, such as primary care databases, but should utilise appropriate methods (e.g., self-report, hospital episode data) to collect comprehensive data reflective of all costs incurred [14].

### 2.4. Time Preference 

An individual’s time preference represents the extent to which they are willing to trade between short-term costs and/or benefits and long-term costs and/or benefits, attributable with a health-related behaviour, such as the consumption of antimicrobials. The time preference rate (discount rate) quantifies the difference between the perceived value of future outcomes relative to more immediate ones. Individuals with lower time preference rates have a higher value for future utility and therefore discount the future less; and conversely, individuals with higher time preference rates have a lower value for future utility and so require greater future reward for their behaviour. Time preference rates for health are typically between 3–6% per annum [30]. In the UK a consistent societal discount rate of 3.5% per annum is applied as recommended in guidance issued by HM Treasury on how to appraise policies, programs, and projects [31]. Variation in the choice of discount rate has a marked effect on studies with long time horizons and is therefore an important consideration for evaluations of strategies aimed to contain resistance—an issue defined by long-term impacts. Given the future value of antibiotics, a lower (zero or perhaps even negative in some circumstances) discount rate may be more relevant for health outcomes [32]. This issue also applies to vaccination, where differential discounting in model-based cost-effectiveness evaluations is being explored [33]. Empirical evidence on time preference rates can be estimated using stated preference techniques that rely on hypothetical scenarios [34]. Participants are typically required to imagine a health state and choose between future outcomes related to that hypothetical health state; this method would generate empirical evidence on individuals time preferences to inform future economic evaluations. A further challenge that will need to be addressed is that in the context of antimicrobial resistance, the future health state to be valued is not that of the respondent but is more likely to be the future health of others. Sensitive analysis using a range of discount rates is recommended.

## 3. Conclusions

Ascertaining the most clinically and cost-effective interventions and services to reduce antimicrobial resistance will require a multifaceted approach, from incentivizing pharmaceutical companies to co-production of patient-centred acceptable interventions that maximise the utility of both patients and society. Economic evidence is central to understanding the value of competing strategies to lessen the probability of resistance development, although the application of such methods poses several methodological challenges. When assessment and interpreting the results of existing economic evaluations of health care interventions in the context of antimicrobial resistance, careful consideration is required, of the perspective, costs, and externality effects (impacts on other patients) of future reductions in antimicrobial effectiveness. We have presented just some of the challenges of accurately estimating the cost-effectiveness of interventions to tackle antimicrobial resistance. Further research is required to capture the effects of antimicrobial resistance in economic evaluations to reflect the value of preserving effective medicines for future use. This requires appropriate consideration of the wider externalities and methodological approaches which differ from standard “reference” case of NICE and other HTA organizations.

## Figures and Tables

**Table 1:** Examples of challenges for the economic evaluation of health care strategies to contain antimicrobial resistance.

Item	Example of Challenges	Recommendations
Population	Population extends beyond those receiving the intervention. This is also likely to extend across health technology agency (HTA) boundaries.	Where appropriate, extend the population beyond the cohort receiving the intervention and consider other/future patients who become infected by a resistant pathogen, or who have not experienced resistant infection but receive alternative agents due to increased resistance of common pathogens.
Clinical	Adequate measurement of the expected rate of growth of antimicrobial resistance and associated outcomes over time. Clinical parameters in the present are more easily captured than those associated with future global consequences.	Use both empirical data and secondary data to forecast long-term clinical consequences. Ensure appropriate assessment of uncertainty.
Costs	Resource implications most likely to be short-term. Difficult to capture long-term resource use and the cost of negative externalities. Cost of health care intervention impacts different budgets to the return, e.g., primary care cost in short-term, for long-term secondary care gains.	Application of robust resource use data collection methods [14]. Include costs of treating patients not receiving the intervention (see population). Use threshold analysis as an alternative to specifying attaching an actual cost to antimicrobial resistance.
Health outcomes	Health states associated with acute infection may be perceived as transient, which limits the validity of trade-off exercises typically used for utility valuation. Utility measures, such as the EQ-5D, measure health “today” and fail to capture the value (utility) associated with future health gains.	Cautious interpretation of quality-adjusted-life year (QALY) gains. Consider alternative or multiple frameworks for analysis, e.g., disability-adjusted-life year (DALYs) to assess global burden, cost-benefit analysis, using contingent valuation.Where appropriate include the disutility for patients with resistant infection and the disutility of alternative agents.
Perspective	Economic evaluations of health care intervention are often restricted to direct health effects and costs with the health technology program considering the evidence. Antimicrobial resistance is a societal issue and extends beyond individual HTA jurisdictions.	Consider a societal perspective to reflect the true range of costs and outcomes. Acknowledge the limitations of HTA by individual agencies.
Time horizon	Evaluations often adopt inadequate time horizons. Time preference may be paradoxical for antimicrobial consumption. Costs and outcomes extend to future generations.	Adopt a lifetime horizon of analysis, use appropriate discounting rates, and conduct empirical research on time preferences for antimicrobial preferences.
